# Human age reversal: Fact or fiction?

**DOI:** 10.1111/acel.13664

**Published:** 2022-07-02

**Authors:** Adiv A. Johnson, Bradley W. English, Maxim N. Shokhirev, David A. Sinclair, Trinna L. Cuellar

**Affiliations:** ^1^ Longevity Sciences, Inc. (dba Tally Health) Greenwich Connecticut USA; ^2^ Blavatnik Institute, Department of Genetics, Paul F. Glenn Center for Biology of Aging Research Harvard Medical School Boston Massachusetts USA

**Keywords:** aging clock, biological age, epigenetic age, healthspan, lifespan, longevity, machine learning, mortality

## Abstract

Although chronological age correlates with various age‐related diseases and conditions, it does not adequately reflect an individual's functional capacity, well‐being, or mortality risk. In contrast, biological age provides information about overall health and indicates how rapidly or slowly a person is aging. Estimates of biological age are thought to be provided by aging clocks, which are computational models (e.g., elastic net) that use a set of inputs (e.g., DNA methylation sites) to make a prediction. In the past decade, aging clock studies have shown that several age‐related diseases, social variables, and mental health conditions associate with an increase in predicted biological age relative to chronological age. This phenomenon of age acceleration is linked to a higher risk of premature mortality. More recent research has demonstrated that predicted biological age is sensitive to specific interventions. Human trials have reported that caloric restriction, a plant‐based diet, lifestyle changes involving exercise, a drug regime including metformin, and vitamin D3 supplementation are all capable of slowing down or reversing an aging clock. Non‐interventional studies have connected high‐quality sleep, physical activity, a healthy diet, and other factors to age deceleration. Specific molecules have been associated with the reduction or reversal of predicted biological age, such as the antihypertensive drug doxazosin or the metabolite alpha‐ketoglutarate. Although rigorous clinical trials are needed to validate these initial findings, existing data suggest that aging clocks are malleable in humans. Additional research is warranted to better understand these computational models and the clinical significance of lowering or reversing their outputs.

## Graphical abstract

Aging is concomitant with a slew of molecular changes, including significant shifts in the epigenome. These age‐related alterations can be used to construct aging clocks, which are computational models thought to predict biological age. The delta between predicted biological age and chronological age correlates with disease burden, mortality, and health. A growing body of evidence indicates that aging clocks are sensitive to health‐promoting (left) and deleterious (right) interventions.

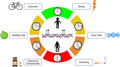

## 1 INTRODUCTION

In 1974, Dr. Werner Ries wrote that the ability to accurately predict biological age (BA) would be of major importance for geriatrics and that a useful predictor would be quantitative, non‐invasive, and reflect human functional capacity (Ries, 1974). Although BA is an abstract concept, it is one that makes intuitive sense and helps explain why different individuals exhibit disparate aging trajectories. It additionally allows for differentiation between people that have an equivalent chronological age (CA). Over the next several decades, multiple attempts were made to quantify this elusive metric. For example, 24 age‐related variables were transformed into BA scores by Borkan and Norris in 1980. Individuals with a higher BA score were estimated to look older by physicians and had a higher risk of mortality (Borkan & Norris, 1980). Subsequent age predictors were created using physiological variables (Dubina et al., 1984), fitness test results (Lee et al., 1996), visual estimation (Olde Rikkert, 1999), frailty index scores (Goggins et al., 2005), physical and biochemical parameters (Bae et al., 2008), and answers to the work ability index (Cho et al., 2010). For all of these models, the correlation between predicted BA and CA varied based on the number of inputs and the specific population being measured. For example, the best‐performing predictors developed by Bae et al had R^2^ values of 0.66 in women and 0.62 in men (Bae et al., 2008).

Following these and other important articles, a pivotal age quantification study emerged from the laboratory of Dr. Eric Vilain in 2011. In this work, Bocklandt et al identified a set of CpG sites whose methylation status correlated remarkably with CA in different datasets. The authors went on to create a multivariate regression model which utilized the methylation status of three cytosines to measure age in saliva with a Pearson correlation of 0.87 and an average error of 3.5 years. Two of these CpGs were associated with the genes *EDARADD* and *ELN* and trended towards demethylation with age. The remaining DNA methylation site was linked to *NPTX2* and showed an age‐dependent trend towards hypermethylation (Bocklandt et al., 2011). Dr. Steve Horvath, who was one of the authors in this study, built upon this work to generate a seminal paper in 2013. Horvath used the elastic net regression model on a large body of methylomic data to identify 353 CpGs that could accurately estimate age in diverse sample types, including whole blood, saliva, buccal cells, and dermal fibroblasts. Horvath dubbed his pan‐tissue model an “epigenetic clock” and used it to show that epigenetic age was significantly elevated in cancer tissue. This phenomenon of age acceleration, defined here as a higher predicted age relative to CA, was pronounced in breast cancer samples harboring mutations in the steroid receptor genes *ESR1* and *PGR* (Horvath, 2013). Earlier that same year, Hannum et al used elastic net and methylomic data to estimate age in whole blood. This clock utilized 71 methylation markers as inputs and predicted that men epigenetically age at a faster rate than women. By making tissue‐specific adjustments to the model, the authors additionally detected age acceleration in cancer samples (Hannum et al., 2013).

Since these pioneering publications by Horvath (Horvath, 2013) and Hannum et al (Hannum et al., 2013), an inordinate amount of progress has been made in the aging clock field, which has also been referred to as biohorology (Galkin et al., 2020). The most common aging clocks use a machine learning model in conjunction with a set of CpG inputs. While less common, other clocks have been created using RNA (Mamoshina et al., 2018), proteins (Enroth et al., 2015), and metabolites (Robinson et al., 2020). The biomedical relevance of these models has been demonstrated by their ability to capture differences in human health. For example, patients with Alzheimer's disease (M. E. Levine et al., 2018), Parkinson's disease (Paul et al., 2021), osteoarthritis (Vidal‐Bralo et al., 2016), obesity (Horvath et al., 2014), coronary heart disease (Roetker et al., 2018), and the premature aging disease Werner syndrome (Maierhofer et al., 2017) have all been reported to exhibit age acceleration. Age acceleration has also been linked to diverse factors, including cigarette smoking (Wu et al., 2019), bipolar disorder (Fries et al., 2020), COVID‐19 infection (Cao et al., 2022), and self‐assessed social status (Hamlat et al., 2022). Importantly, age acceleration correlates with premature mortality (Perna et al., 2016) and functional capacity. For example, the mortality predictor and epigenetic aging clock GrimAge (A. T. Lu, Quach, et al., 2019) was recently shown to correlate with reaction time, cognitive function, polypharmacy, frailty, and walking speed in older adults (McCrory et al., 2021). The ability of these models to predict diverse age‐related outcomes suggests that they can provide insights into BA. As such, we subsequently refer to the outputs of aging clocks as BA. Considerations surrounding this labeling are discussed in the “Outstanding Questions and Limitations in the Field” section.

On the whole, a considerable amount of research in the biohorology field has shown that specific diseases and factors are linked to age acceleration. In comparison, only a small number of aging clock studies have connected a particular intervention or variable to a decrease in BA. In this review, we focus on the latter and highlight preliminary evidence suggesting that aging clocks can be slowed and reversed in humans.

### 1.1 Machine learning and age prediction

Since artificial intelligence is often conceptualized as a mysterious dark box (Castelvecchi, 2016) and lies at the heart of aging clocks, we will briefly introduce how machine learning models identify and utilize features for age prediction. We refer the interested reader to more in‐depth reviews of this topic (Galkin et al., 2020; Zhavoronkov & Mamoshina, 2019).

Typically, the first step is selecting the best set of inputs that can be combined to estimate CA. Genome‐wide measurements are usually obtained using array‐based or high‐throughput sequencing assays, resulting in measurements of thousands of proteins (Tin et al., 2019), tens of thousands of genes (Duggan et al., 1999), or even millions of DNA methylation sites (Zhang & Jeltsch, 2010) for each sample. For array‐based methylomics, the Infinium HumanMethylation450 and MethylationEPIC chips offered by Illumina are commonly utilized (Bell et al., 2019). However, the limited number of age values results in more inputs than there are measured outcomes. This means that age prediction is inherently an underdetermined problem, or that many different sets of inputs can be used (Porter et al., 2021). Therefore, a feature selection step is needed to identify the most informative input values. There are many ways to explicitly filter the inputs. For example, inputs that have low variance across age or are highly correlated to other inputs can be removed. In addition, inputs that have low read coverage can be omitted to minimize noise. Next, inputs are often transformed prior to model training to remove bias. Common transformations include (1) normalization to balance the importance of highly expressed inputs with, for example, a log transformation, (2) confounding factor correction to remove batch effects (Leek et al., 2010) or account for cell type composition (Lowe & Rakyan, 2014), and (3) dimensionality reduction using linear decompositions such as principal component analysis (Higgins‐Chen et al., 2021; M. Levine et al., 2020). The resulting datasets can then be used to train age prediction models.

Many models exist for mapping inputs into an age readout, including random forest (Schultz et al., 2020), elastic net (Horvath, 2013), and least absolute shrinkage and selection operator (LASSO) (Lehallier et al., 2020). There are others (Galkin et al., 2020; Zhavoronkov & Mamoshina, 2019) that vary in their structure, interpretability, and assumptions. In the case of a continuous variable age readout, a regression model is needed (modeling discrete outcomes requires a different type of model called a classifier). While we previously found random forest to be especially adept at predicting transcriptomic age (Shokhirev & Johnson, 2021), elastic net and LASSO models are simpler to interpret and work well for epigenetic age prediction (Bell et al., 2019). Importantly, some models include implicit feature selection as part of their training and structure, and are appropriate for underdetermined problems. For example, a penalty is added to LASSO regression models that depends on the total magnitude of the weights. During training, some weights are adjusted down to zero, implicitly removing less informative inputs from the model. In other words, there is no one answer and the specific model chosen must be justified in the context of the data structure, interpretability, and performance.

After a model is selected and inputs are preprocessed, model training takes place. Each model has its own algorithm for adjusting internal parameters to produce the most optimal prediction. In random forest models, random decision trees are built from a subset of the possible inputs, and the trees combine their prediction as an ensemble called a forest (X. Chen & Ishwaran, 2012). In the case of age prediction (Shokhirev & Johnson, 2021), decision trees are built hierarchically from the root down by finding thresholds in input values that result in the lowest error compared to the average CA of the samples in each partition. Additional branches are added to further partition the samples until the leaves represent a small number of samples. Since each decision tree is built using a subset of the data and the answer is compiled from all trees in a forest, this helps to minimize overfitting and promote generalizability. Regardless of the model used, the problem of generalizability is approached by splitting the data into training and test sets. This helps ensure that the model does not have a chance to memorize the data.

**FIGURE 1:**
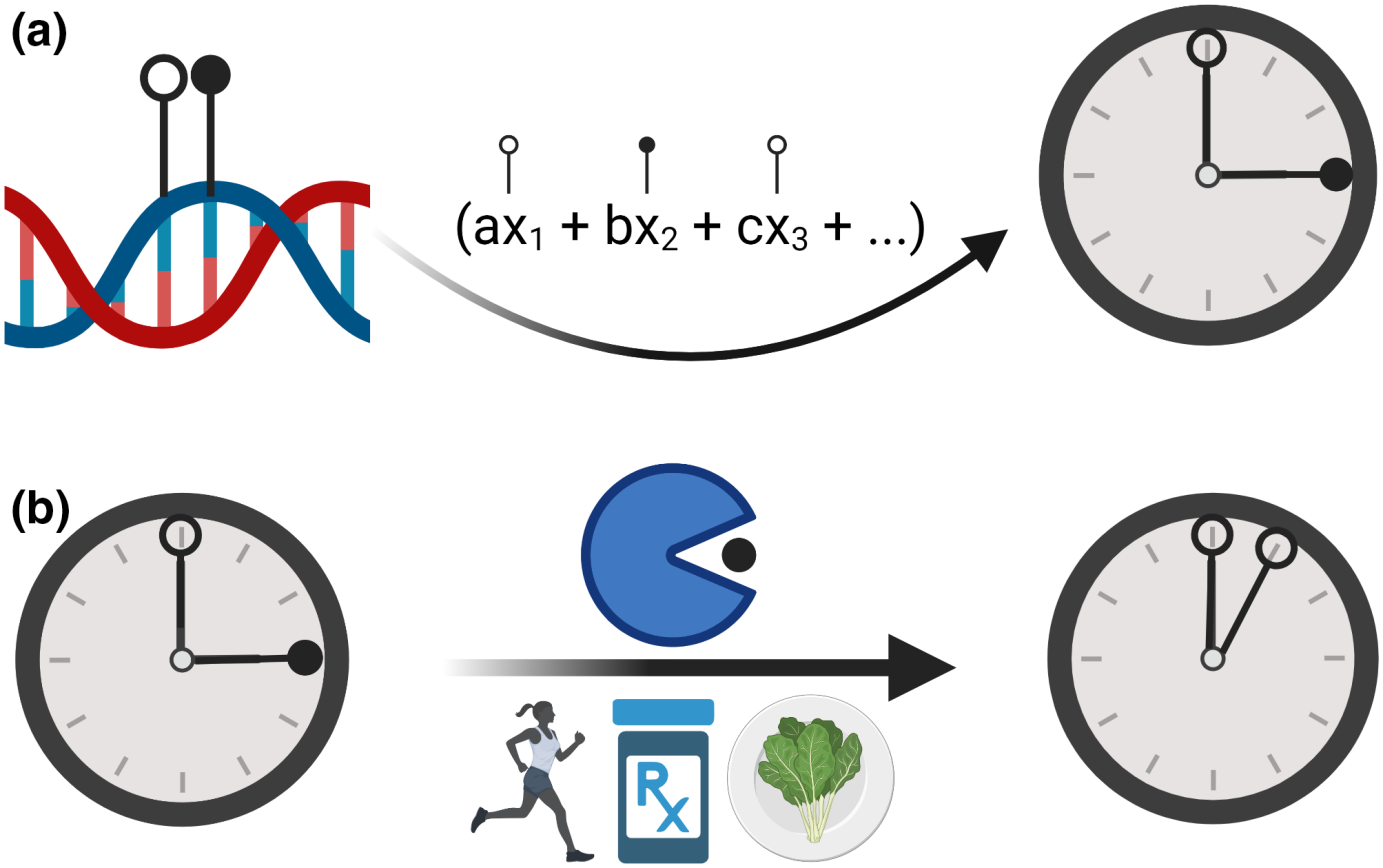
Aging clocks are targetable. (a) With age, the methylome undergoes significant changes characterized by aberrant hypermethylation and hypomethylation. These age‐associated epigenetic changes serve as the basis for epigenetic aging clocks that are thought to measure biological age. (b) Existing evidence suggests that aging clocks are malleable and can be slowed or reversed in response to various interventions, such as caloric restriction, a plant‐based diet, drugs, or lifestyle change involving physical activity

When used correctly, these machine learning models (Figure 1a) are accurate and able to make predictions in new data of the same type. These clocks can even be dissected to gain insights into aging mechanisms (Raj & Horvath, 2020) and identify potential drug targets relevant to age‐related disease (Johnson et al., 2021). These models can be conceptualized as representing various mathematical windows into BA as a function of distinct biological readouts. The predicted age, which is a proxy for BA and inevitably deviates from CA, can be a valuable tool for predicting age‐related health outcomes.

### 1.2 Aging clocks are targetable in complex animals

Ever since Dr. Cynthia Kenyon's discovery that longevity in *Caenorhabditis elegans* could be doubled by introducing a single mutation in the insulin receptor‐like gene *daf‐2* (Kenyon et al., 1993), a slew of research has demonstrated that different genetic (Brown‐Borg et al., 1996), dietary (Mattison et al., 2017), pharmacological (Bitto et al., 2016), and behavioral (Nilsson et al., 2019) interventions are capable of extending lifespan and/or improving healthspan in complex model organisms. If an aging clock truly reflects an individual's unique aging rate and overall health, we would expect its output to be significantly lowered by established aging interventions.

Arguably, the most evolutionarily conserved life extension strategy is caloric restriction without malnutrition (Fontana & Partridge, 2015). Decreasing dietary intake has been reported to prolong lifespan in diverse organisms, including yeast (S. J. Lin et al., 2004), mosquitoes (Joy et al., 2010), fish (Terzibasi et al., 2009), and monkeys (Mattison et al., 2017). Indicative of an ability to measure this longevity effect, different research groups have found that restricting calories lowers epigenetic age in mice and rats (M. Levine et al., 2020; Meer et al., 2018; Minteer et al., 2022; Petkovich et al., 2017; Thompson et al., 2018; Wang et al., 2017). A related dietary intervention is methionine restriction, which lengthens life, enhances stress resistance, and augments health in mice (Miller et al., 2005). Using frailty index scores as inputs, Schultz et al used random forest machine learning to create models that estimate BA or time‐to‐death. Mice that were subjected to methionine restriction had lower frailty index scores and were predicted to be both younger and longer‐lived (Schultz et al., 2020). Although non‐dietary, a distinct lifestyle intervention of exercise reduces the epigenetic age of skeletal muscle in mice aged 22–24 months. Older mice that engaged in voluntary progressive weighted wheel running were epigenetically ~8 weeks younger than their sedentary counterparts (Murach et al., 2022).

Different pharmacological treatments can also target aging clocks in mice. In the same study by Schultz et al, the authors treated animals with the angiotensin converting enzyme inhibitor enalapril. Like methionine restriction, enalapril decreased frailty index scores and lowered BA. Enalapril did not significantly impact predicted time‐to‐death, however, potentially suggesting that it improved health without affecting lifespan (Schultz et al., 2020). In aged female mice, Florian et al showed that inhibiting Cdc42 with a molecule termed CASIN elongated lifespan, decreased epigenetic age, and reset the expression of inflammatory cytokines in serum to a youthful level (Florian et al., 2020). Age deceleration – defined here as a lower BA relative to CA—in mice was observed in response to treatment with rapamycin (Wang et al., 2017), an immunosuppressant drug with a well‐established ability to boost longevity (Bitto et al., 2016). Intriguingly, rapamycin was not associated with a significantly reduced epigenetic age in a separate study done in marmoset monkeys (Horvath et al., 2021). Whether or not this is due to the concentration of rapamycin, treatment duration, the specific clocks used, or differences between mice and marmosets remains to be determined. One explanation for the differential results may be the disparate tissues analyzed. Measurements were made in liver and blood for mice (Wang et al., 2017) and marmosets (Horvath et al., 2021), respectively.

Genetic mutations or gene therapy are discrete paths towards slowing an animal's molecular clock. For example, long‐lived mice that are deficient in growth hormone exhibit a decrease in epigenetic age. This includes mice lacking *Ghr* as well as animals carrying mutations in either *Prop1* or *Pou1f1* (Meer et al., 2018; Petkovich et al., 2017; Wang et al., 2017). Separate work demonstrated that ectopically expressing the Yamanaka factors *Oct4*, *Sox2*, and *Klf4* using adeno‐associated viral vectors restored vision in older mice and reversed epigenetic age in retinal tissue. This reprogramming approach was also able to recover eyesight in a mouse model of glaucoma (Y. Lu et al., 2020). More recent research from the laboratory of Dr. Juan Carlos Izpisua Belmonte showed that epigenetic age in skin and kidney was reverted in response to long‐term partial reprogramming in wild‐type mice. This reversion was concomitant with improved wound healing and a reduced inflammatory signature in skin (Browder et al., 2022).

These data cumulatively indicate that aging clocks are sensitive to pro‐longevity interventions in complex model organisms.

### 1.3 Interventions that turn back aging clocks in humans

In 2015, the results from the CALERIE (Comprehensive Assessment of Long term Effects of Reducing Intake of Energy) trial were published (Ravussin et al., 2015). 220 non‐obese adults were randomized and placed on either a 25% caloric restriction or ad‐libitum diet for 2 years. Although the mean caloric restriction practically achieved was 11.7%, this was sufficient to promote weight loss, induce a decrease in circulating tumor necrosis factor‐α, and cause a reduction in cardiometabolic risk factors (Ravussin et al., 2015). Using the Klemera‐Doubal Method (Klemera & Doubal, 2006) and clinical biomarker data collected during this trial, Belsky et al subsequently estimated the BA of individuals in both trial arms. Ad‐libitum and calorically restricted participants exhibited an annual BA change of 0.71 and 0.11 years, respectively. This delta of 0.6 years was significantly different and, in the ad‐libitum group, BA was significantly higher after 2 years. Consistent with a deceleration in aging, BA was statistically comparable after 2 years in calorically restricted subjects (Belsky et al., 2017).

Other human trials have similarly reported that a dietary intervention can affect an aging clock. In work conducted by Gensous et al, 120 healthy elderly Italian and Polish subjects (60 from each country) were subjected to a Mediterranean‐like diet for a duration of 1 year. Horvath's classical model was used to measure epigenetic age in whole blood before and after the 12‐month nutritional intervention. Although the results varied based on sex and country of residence, the delta between BA and CA (∆age) was reduced by 0.84 years in Polish subjects. In Polish women, ∆age decreased by −1.47 years. These individuals exhibited a ∆age that was lower than it was pre‐intervention a year prior. The authors theorize that differences between groups may be due to cultural or social factors (Gensous et al., 2020). For example, the similarity of a pre‐intervention diet to the Mediterranean diet could have influenced the results. Fiorito et al analogously found that an altered diet could reduce epigenetic age in a cohort of 219 healthy, postmenopausal women. After 24 months of adopting a diet based on the consumption of plant foods, ∆age was 0.66 years lower relative to controls (Fiorito et al., 2021). Combination therapies including diet and exercise have also been reported to lower BA in healthy subjects (Fitzgerald et al., 2021) and in individuals with obesity or dyslipidemia (Yaskolka Meir et al., 2021). In one of these studies, a comprehensive lifestyle intervention led to a significant decrease in Horvath's classical clock relative to controls after a short period of 8 weeks. Although it only trended towards statistical significance (*p* = 0.066), subjects in the treatment group were predicted to have an epigenetic age that was 1.96 years lower than when they started the study (Fitzgerald et al., 2021). In a separate cohort of patients with severe obesity, a decrease in epigenetic ∆age was observed 12 months after bariatric surgery (Fraszczyk et al., 2020).

Early‐stage data indicate that an individual's aging clock can also be targeted pharmacologically. A pilot, non‐placebo‐controlled study by Fahy et al gave metformin, growth hormone, and dehydroepiandrosterone to 10 healthy adult men aged 51–65 years. The authors theorized that the diabetic drug metformin would help counteract the deleterious effects of growth hormone, which can induce hyperinsulinemia. Concomitant with immunological changes, epigenetic age was reversed after a year of treatment. While the results differed based on the specific model used, subjects had an epigenetic age that was 2.16 years younger than when they began treatment 12 months earlier according to the GrimAge clock (Fahy et al., 2019). A separate randomized, placebo‐controlled clinical trial investigated the effects of vitamin D3 supplementation in obese/overweight individuals with low vitamin D status. Using either the Horvath or Hannum clock, the authors found that vitamin D3 supplementation respectively decreased epigenetic age by 1.85 or 1.9 years compared to placebo (L. Chen et al., 2019). Drug treatment additionally influences epigenetic age in patients with HIV. 96 weeks of anti‐retroviral therapy led to a 3.6 year decrease in ∆age (Esteban‐Cantos et al., 2021). Although it wasn't an interventional trial, a recent study reported that transient reprogramming reversed a transcriptomic aging clock by approximately 30 years in vitro in human dermal fibroblasts (Gill et al., 2022).

It is important to note that some aging clock trials have reported negative results. For example, Nwanaji‐Enwerem et al performed a post‐hoc analysis of a placebo‐controlled, randomized control trial involving 192 overweight or obese breast cancer survivors. This trial lasted for 6 months and included four intervention arms: placebo, placebo with weight loss, metformin, and metformin with weight loss. Using various clocks, the authors found that epigenetic age was not significantly altered in any group (Nwanaji‐Enwerem et al., 2021). In an independent observational study, metformin was not linked to a delay in epigenetic age (Quach et al., 2017). This is intriguing given that metformin is associated with protection against various age‐related diseases (Barzilai et al., 2016) and reduced GrimAge when used alongside growth hormone and dehydroepiandrosterone (Fahy et al., 2019). Future research efforts are warranted to better understand the relationship between metformin and aging clocks. In a separate placebo‐controlled clinical trial involving 1470 subjects, daily consumption of 5 mg folic acid and 30 mg elemental zinc for 6 months did not influence epigenetic age in sperm (Jenkins et al., 2022). Compared to controls, epigenetic age was similarly unimpacted by 60 g/day of mixed nuts in a 14‐week trial involving 72 participants (Salas‐Huetos et al., 2021). In the aforementioned study by Fiorito et al, exercise did not significantly impact BA. It did, however, correct aberrant methylation patterns in pathways related to cancer (Fiorito et al., 2021). Since not all trials are pre‐registered, it is possible that there are other unpublished studies which found negative results.

In each of the studies reporting a significant reduction in BA (Table 1), an aging clock was used to make a prediction before and after an intervention. The findings from these preliminary trials collectively suggest that it is possible to intervene and decrease BA in humans (Figure 1b). Although exciting, many of these trials were fairly short‐term and used a small number of subjects. Larger‐scale, placebo‐controlled studies are warranted to validate these results, perform measurements over longer time courses, and determine the extent to which BA can be reduced.

**TABLE 1:** Dietary, lifestyle, and pharmacological interventions reported to slow or reverse an aging clock in humans

Intervention	Result	Aging clock used	Subject #	Health status	Age information (years)	Study reference
25% caloric restriction	Compared to the ad‐libitum group, the caloric restriction group was **0.6** years younger after **24 months**	Klemera‐Doubal Method (Klemera & Doubal, 2006)	220	Non‐obese	21–50	Belsky et al. (2017)
Metformin, growth hormone, and dehydroepiandrosterone	Compared to baseline, epigenetic age was decreased by **2.16** years after **12 months**	GrimAge (A. T. Lu, Quach, et al., 2019)	10	Healthy	51–65	Fahy et al. (2019)
Vitamin D3	2000 IU/day of vitamin D3 for **16 weeks** decreased epigenetic age by **1.9** years compared to placebo	Hannum (Hannum et al., 2013)	51	Overweight or obese with low vitamin D status	26.1 ± 9.3	L. Chen et al. (2019)
Bariatric surgery	**12 months** post‐surgery, ∆age decreased by **0.92** years	Horvath (Horvath, 2013)	40	Severe obesity	45.1 ± 8.06	Fraszczyk et al. (2020)
Mediterranean‐like diet	In Polish subjects, ∆age was **0.84** years less than it was pre‐intervention **12 months** prior	Horvath (Horvath, 2013)	120	Healthy	65–79	Gensous et al. (2020)
Antiretroviral therapy	Drug treatment for **96 weeks** decreased ∆age by **3.6** years	PhenoAge (M. E. Levine et al., 2018)	168	HIV	30–46	Esteban‐Cantos et al. (2021)
Plant‐based diet	Relative to controls, ∆age was reduced by **0.66** years after **24 months**	GrimAge (A. T. Lu, Quach, et al., 2019)	219	Healthy	50–69	Fiorito et al. (2021)
Plant‐centered diet, supplements, exercise, sleep, and stress management	Compared to controls, an **8‐week** intervention decreased epigenetic age by **3.23** years	Horvath (2013)	43	Healthy	50–72	Fitzgerald et al. (2021)
Diet (low‐fat or Mediterranean/low‐carbohydrate) and physical activity	Compared to individuals that failed to lose weight, subjects that successfully lost weight were **0.5** years younger after **18 months**	J. Li et al. (2018)	120	Obesity or dyslipidemia	48.6 ± 9.3	Yaskolka Meir et al. (2021)

### 1.4 Factors associated with a slower aging clock in humans

A multitude of factors have been shown to associate with human age deceleration (Table 2). A study performed by Quach et al looked at cross‐sectional data from 4575 individuals spanning two different cohorts. Using metrics of ∆age, they identified several variables to be significantly correlated with slower epigenetic aging. These factors include fish intake, levels of blood markers for fruit/vegetable consumption, physical activity, education, and income (Quach et al., 2017). Subsequent work by Levine et al constructed a new epigenetic clock called PhenoAge that was optimized to predict mortality, healthspan, and physical functioning. PhenoAge measurements corroborated that education, income, exercise, and markers of fruit/vegetable consumption are linked with a lower epigenetic age (M. E. Levine et al., 2018). We similarly found a connection between increased physical activity and age deceleration using a plasma proteomic clock. In collaboration with Drs. Benoit Lehallier and Tony‐Wyss Coray, we applied this model in 47 healthy adults that were either sedentary or engaged in frequent aerobic exercise. The difference in proteomic age was significant, with aerobic exercise‐trained individuals estimated to be 5.43 years younger (Lehallier et al., 2020). Other groups have likewise connected dietary factors, physical activity, and other lifestyle choices to a slower aging clock (Table 2). Interesting examples to highlight include omega‐3 supplementation (A. T. Lu, Quach, et al., 2019), light alcohol consumption (Liang et al., 2022), moderate coffee consumption (Enroth et al., 2015), good sleep quality (Gao et al., 2022), vitamin D supplementation (Vetter et al., 2022), and Mediterranean diet adherence (Esposito et al., 2022).

**TABLE 2:** Factors associated with a slower aging clock in humans

Factor(s)	Aging clock(s) used	Cohort size	Age information (years)	Tissue/data analyzed	Study reference
Fatty fish consumption, coffee consumption, exercise	Enroth et al. (2015)	976	14–94	Plasma	Enroth et al. (2015)
Smoking cessation	Horvath (2013) and Hannum et al. (2013)	22	46.77 ± 6.99	Blood	Lei et al. (2017)
Poultry intake, fish intake, markers of vegetable/fruit consumption, education, income, exercise, alcohol consumption	Horvath (2013) and Hannum et al. (2013)	4575	30–100	Blood	Quach et al. (2017)
Markers of vegetable/fruit consumption, nut consumption, education, income, exercise, alcohol consumption	PhenoAge (M. E. Levine et al., 2018)	4207	50–79	Blood	M. E. Levine et al. (2018)
Omega‐3 supplementation, carbohydrate intake, dairy intake, whole grain intake, markers of vegetable/fruit consumption, education, income, exercise, alcohol consumption	GrimAge (A. T. Lu, Quach, et al., 2019)	2174	59–73^a^	Blood	A. T. Lu, Quach, et al. (2019)
Aerobic exercise	Lehallier (Lehallier et al., 2020)	47	19–77	Plasma	Lehallier et al. (2020)
Calcium alpha‐ketoglutarate	TruAge (Demidenko et al., 2021)	42	43–72	Saliva	Demidenko et al. (2021)
Leisure‐time physical activity	GrimAge (A. T. Lu, Quach, et al., 2019)	1040	21–74	Blood	Kankaanpää et al. (2021)
Doxazosin, fiber intake, magnesium intake, vitamin E intake	MoveAge (McIntyre et al., 2021)	5139	18–85+	Accelerometer data	McIntyre et al. (2021)
Lifestyle factors, including physical activity, intake of vegetables and fruits, and moderate drinking	Li (J. Li et al., 2018)	286	48.9 ± 10.6	Blood	Peng et al. (2021)
Cardiovascular health factors, including diet, smoking status, and physical activity	Horvath (Horvath, 2013) and Hannum (Hannum et al., 2013)	2170	64.19 ± 7.06	Blood	Pottinger et al. (2021)
Mediterranean diet, Dietary Approaches to Stop Hypertension diet	Esposito (Esposito et al., 2022)	4510	≥ 35	Blood	Esposito et al. (2022)
Sleep quality	Klemera‐Doubal Method (Klemera & Doubal, 2006) and PhenoAge (M. E. Levine et al., 2018)	363,886	56.5 ± 8.1	Blood	Gao et al. (2022)
Higher diet quality	DunedinPoAm (Belsky et al., 2020), GrimAge (A. T. Lu, Quach, et al., 2019), and PhenoAge (M. E. Levine et al., 2018)	1995	67 ± 9	Blood	Y. Kim et al. (2022)
Higher diet quality	Hannum (Hannum et al., 2013), PhenoAge (M. E. Levine et al., 2018), and GrimAge (A. T. Lu, Quach, et al., 2019)	2694	56 ± 9	Blood	Kresovich et al. (2022)
Light alcohol consumption	MonoDNAmAge (Liang et al., 2022), Horvath (Horvath, 2013), Hannum (Hannum et al., 2013), PhenoAge (M. E. Levine et al., 2018), and GrimAge (A. T. Lu, Quach, et al., 2019)	2242	18–83	Monocytes, blood, and peripheral blood mononuclear cells	Liang et al. (2022)
Serum zinc levels	Horvath (2013)	10	37.83 ± 12.05	Blood leukocytes	Noronha et al. (2022)
Vitamin D supplementation	Horvath (2013) and Vetter et al. (2019)	1036	68.28 ± 3.49	Blood	Vetter et al. (2022)

^a^ Self‐reported omega‐3 intake data was available for 2174 members of a larger cohort composed of 2356 people. The age range provided is for the full cohort (*n* = 2356).

A smaller set of studies have found a specific molecule or drug to be associated with a decrease in BA. In a recent retrospective analysis, epigenetic age was calculated in 42 subjects taking a supplement containing 1000 mg of calcium alpha‐ketoglutarate for an average period of 7 months. A novel clock predicted that these individuals were 8 years younger post‐supplementation (Demidenko et al., 2021). Although a placebo‐controlled trial is needed to validate this finding and determine if clinically meaningful changes are concomitant with such a drastic reduction in epigenetic age, they are interesting given that calcium alpha‐ketoglutarate extends lifespan and improves health in mice (Asadi Shahmirzadi et al., 2020). Using a rather innovative model based on wearable device movement data, McIntyre et al linked the FDA‐approved, antihypertensive drug doxazosin to age deceleration. In the same study, the authors demonstrate that doxazosin elongates both lifespan and healthspan in nematode worms. The dietary intake of fiber, magnesium, and vitamin E was also associated with decelerated aging (McIntyre et al., 2021). Since a large number of molecules have been reported to enhance lifespan and/or healthspan in animal models (Tacutu et al., 2018), future research efforts should assess whether or not any of these compounds can safely influence aging clocks in humans.

### 1.5 Outstanding questions and limitations in the field

In this work, we refer to the measurement made by an aging clock as BA given that the disparity between BA and CA significantly correlates with age‐related health outcomes such as mortality (Perna et al., 2016) and disease burden (Hillary et al., 2020). Whether or not the metric provided by an aging clock truly represents BA is, however, debatable. Ultimately, these clocks make a calculation based on a set of inputs, which are typically molecular in nature and predictably vary with age in a population. In the case of epigenetic models, the methylation status (i.e., methylated or demethylated) of CpGs is utilized. If an intervention decreases the number outputted by an epigenetic clock, this means that the status of specific DNA methylation sites resembles that of a younger individual. While such a change may indicate that an individual has become biologically younger, it is feasible that a more youthful epigenetic signature can be induced irrespective of BA. One way to explore these two possibilities would be to determine if inputs used by aging clocks represent downstream biomarkers or instead causally contribute to age‐related dysfunction. For example, the CpGs cg16867657 and cg21572722 were prioritized by a newer epigenetic aging clock trained using methylomic information shared by Illumina's Infinium HumanMethylation450 and MethylationEPIC arrays (Horvath et al., 2018). Both of these sites become hypermethylated with age and are associated with the gene *ELOVL2*, which encodes for Elongation of very long chain fatty acids protein 2 (UniProt, 2021). In mice, introducing a point mutation in *Elovl2* results in premature visual decline and the early appearance of autofluorescent material typically seen in older animals. In addition, treating mice with the DNA methylation inhibitor 5‐Aza‐2′‐deoxycytidine demethylates the promoter of *Elovl2*, increases gene expression of *Elovl2*, and rescues age‐related visual decline (D. Chen et al., 2020). Additional research is thoroughly warranted to better understand the relationship between BA and aging clocks.

It is also worth noting that not all clocks are equivalently predictive when it comes to aging‐relevant measurements. Indicative of this, GrimAge outperformed the PhenoAge, Horvath, and Hannum clocks when it came to predicting health and mortality in a longitudinal dataset involving 490 older subjects (McCrory et al., 2021). Recent work by Macdonald‐Dunlop et al performed a thorough comparison of 15 different omics‐based clocks. The authors found that, while some clocks correlated well with specific disease risk factors (e.g., systolic blood pressure and cortisol), others were more prognostic of age‐related disease incidence and appeared to better reflect the generalized effects of aging (Macdonald‐Dunlop et al., 2022). Moreover, there is an interesting relationship between accuracy and usability. If a clock becomes overly adept at predicting CA and the average delta between BA and CA is too low, then it will fail to correlate with meaningful health outcomes (Q. Zhang, Vallerga, et al., 2019). In addition, each distinct clock uses a unique set of inputs that correspond to different biological processes. For example, we previously showed that accurate, plasma proteomic clocks could be generated using proteins associated with disparate pathways in the Reactome database, such as the “signal transduction”, “innate immune system”, “extracellular matrix organization”, and “adaptive immune system” pathways (Lehallier et al., 2020). Since aging is a multifarious process characterized by diverse, complex dysfunction (Lopez‐Otin et al., 2013), it is possible that each of these clocks provides a unique window into BA. Indeed, one could imagine that the opacity of these windows varies depending on the quality and type of aging clock. Alternatively, it could be argued that distinct models reflect different aspects of health and biology that correlate with CA. For example, the epigenetic model DNAmTL was trained to predict telomere length. Although DNAmTL correlates with telomere length, CA, and health outcomes, it does not match telomere length in cultured cells. Instead, it appears to capture population doubling as evinced by the finding that cells expressing telomerase exhibit a passage‐dependent reduction in DNAmTL (A. T. Lu, Seeboth, et al., 2019). Thus, DNAmTL appears to be capturing an interesting signal that is distinct from what it was originally trained on.

Another consideration is the relationship between aging clocks and health. Specifically, does a reduction in BA clearly translate to a tangible improvement in well‐being? In each of the human interventional studies summarized in Table 1, there is independent evidence that the intervention itself or an aspect of the intervention promotes health and/or decreases mortality. For example, clinical trials in humans have shown that caloric restriction induces weight loss (Ravussin et al., 2015), improves thymopoiesis (Spadaro et al., 2022), elevates sleep quality (Martin et al., 2016), and attenuates the expression of circulating inflammatory factors (Montefusco et al., 2021). Regarding metformin, existing evidence suggests that this diabetic drug may protect against various age‐related diseases (Barzilai et al., 2016). Indicative of this, a systematic review and meta‐analysis concluded that all‐cause mortality was lower in diabetic, metformin‐users compared to non‐diabetics (Campbell et al., 2017). The Targeting Ageing with Metformin (TAME) trial intends to rigorously test the feasibility and safety of metformin as an aging intervention in older adults (https://www.afar.org/tame‐trial). In a recently published clinical trial involving 25,871 subjects, supplementation with vitamin D3 for 5 years led to a significant, 22% decrease in autoimmune diseases, including the age‐related disease rheumatoid arthritis (Hahn et al., 2022). Moreover, a large‐scale systematic review and meta‐analysis concluded that vitamin D supplementation associates with a reduced risk of cancer mortality (Y. Zhang, Fang, et al., 2019). The Mediterranean diet, which is considered to be plant‐based, attenuates the progression of atherosclerosis to coronary heart disease (Jimenez‐Torres et al., 2021), improves cognitive function in older adults (Valls‐Pedret et al., 2015), and reduces the incidence of major cardiovascular events (Estruch et al., 2018). An ever‐growing body of evidence similarly argues that physical activity and a plant‐rich diet promote healthy aging in humans (H. Kim et al., 2019; Y. H. Lin et al., 2020). Both antiretroviral therapy (Zhao et al., 2018) and gastric bypass surgery (Adams et al., 2007) reduce mortality in patients with HIV or severe obesity, respectively. For the interventional studies summarized in Table 1, the reported reduction in BA may therefore reflect improvements in health and a shift towards a more optimal aging trajectory.

Future trials using aging clocks should also take care to make traditional clinical measurements. Tests that assess functional performance in older adults – such as grip strength, gait speed, the 6‐min walk test, and the timed up‐and‐go test (Patrizio et al., 2021) – are especially valuable. In addition to estimating BA, it would be helpful to measure classical clinical biomarkers that are known to associate with lifespan and healthspan. These include HbA1c, fasting blood glucose, C‐reactive protein, triglycerides, high‐density lipoprotein cholesterol, ApoA1, and total cholesterol (X. Li et al., 2021). Ultimately, the utility of BA being reduced without a concomitant functional improvement and/or a decreased risk of mortality is questionable. Conversely, a reduction in BA that is tethered to a clear enhancement in health and/or longer life is of interest. Long‐term, longitudinal trials in older populations would be exceptionally valuable and offer insight into how a change in BA alters mortality‐risk on an individual level. As more trials are published, we will gain a more thorough understanding of how clinically significant altering an aging clock is. Additional data will also help inform how these computational models compare to existing clinical diagnostics. Since it is possible that the participants drawn to these studies may be uniquely interested in health relative to the general population, placebo controls are especially important. Furthermore, traditional epigenetic aging clocks exhibit technical noise and replicates from the same sample can produce divergent results (McEwen et al., 2018). There are newer methods to control for this (Higgins‐Chen et al., 2021) and this should be considered when deciding which computational model to implement.

Regarding future studies, there are a number of clinical trials that intend to explore whether or not a particular intervention will affect an aging clock. This includes the TRIIM‐X trial (NCT04375657), which is an expansion of the aforementioned metformin, growth hormone, and dehydroepiandrosterone study by Fahy et al (Fahy et al., 2019). Other proposed trials seek to test the effects of tildrakizumab (NCT05110313), a polyphenol‐rich supplement (NCT05234203), a fasting‐mimicking diet in conjunction with a calorie mimetic supplement (NCT04962464), dasatinib and quercetin (NCT04946383), a sleep supplement (NCT04988542), tree nuts and extra virgin olive oil (NCT04361617), and rapamycin (NCT04488601). These and future trials will enhance our collective understanding of what influences BA in humans.

A final point of consideration is the reversal of an aging clock, which has been reported in vivo in mice (Browder et al., 2022; Y. Lu et al., 2020) and in vitro in human cells (Gill et al., 2022) in response to reprogramming. As shown in Table 1, epigenetic age can also be reversed in response to different interventions. Although exciting, these results should be interpreted conservatively given that no intervention has been found to stall aging in any organism. These findings are likely showing that adopting a health‐promoting change – such as transitioning to a healthy diet and increasing recreational physical activity – can reset an individual's aging trajectory. Such changes are of course possible and well‐established. For example, quitting smoking following myocardial infarction leads to a substantial decrease in mortality (Wilson et al., 2000).

## 2 CONCLUDING REMARKS

In addition to being useful research tools, aging clocks have the potential to inform decision‐making and provide personal evidence that a specific change is connected to slower or faster aging. Although the field of biohorology is relatively nascent and still developing, it holds promise for helping people live longer, healthier lives. Preliminary data support this optimism and argue that aging clocks are sensitive to health‐promoting interventions in humans. Future research efforts are warranted to better understand the relationship between these computational models, health, and longevity. Specifically, the clinical significance of slowing or reversing an aging clock needs to be elucidated.

## AUTHOR CONTRIBUTIONS

AAJ participated in design, writing, and editing. BWE generated graphics and helped with editing. MNS assisted with writing and editing. DAS provided resources and oversight. TLC contributed to design and editing.

## CONFLICT OF INTEREST

AAJ, MNS, and TLC are full‐time employees of the biotechnology company Tally Health, Inc. BWE performed consulting work for Tally Health. DAS is a consultant to, inventor of patents licensed to, and in some cases board member and investor in MetroBiotech, Cohbar, Life Biosciences and affiliates, Zymo, EdenRoc Sciences and affiliates, Alterity, InsideTracker, Immetas, Segterra, Galilei Biosciences, and Tally Health. He is also an inventor on patent applications licensed to Bayer Crops, Merck KGaA, and Elysium Health. Additional info can be found at the following link: https://sinclair.hms.harvard.edu/david‐sinclairs‐affiliations. The authors have no other conflicts of interest to declare.

## Data Availability

Data sharing is not applicable to this article as no new data were created or analyzed in this study.
